# Bone marrow aspirate Cit-H3 was identified as a novel biomarker of minimal residual disease in neuroblastoma

**DOI:** 10.1186/s12885-025-15305-3

**Published:** 2025-11-26

**Authors:** Mengxia Zhang, Xiang Wang, Lin Liu, Yuge Ni, Yuanyuan Zhang, Xiaoyan Hu, Chen Huang, Baocheng Gong, Tiantian She, Chong Chen

**Affiliations:** 1 Department of Clinical Laboratory, Tianjin Union Medical Center, The First Affiliated Hospital of Nankai University, Tianjin, China; 2 Department of Medical Technology, Tianjin Medical University, Tianjin, China; 3 Department of Pediatric Oncology, Tianjin Medical University Cancer Institute and Hospital, National Clinical Research Center for Cancer, Tianjin’s Clinical Research Center for Cancer, Tianjin, China

**Keywords:** Neutrophil extracellular traps, Neuroblastoma, Minimal residual disease, Cit-H3, NSE

## Abstract

**Background:**

Neuroblastoma (NB) is the most prevalent extracranial solid tumor in children. Neutrophils release neutrophil extracellular traps (NETs) following intense or prolonged activation. Neutrophil elastase (ELA2) and citrullinated histone H3 (Cit-H3) are specific markers of NETs. Additionally, neuron-specific enolase (NSE) serves as a biomarker and can be used to monitor the condition of NB progression, assess treatment response, and predict recurrence.

**Methods:**

This study employed a retrospective study design, and all samples were collected prior to bone marrow aspiration for diagnosis. A total of 39 serum samples from patients diagnosed with NB by postoperative pathological bone marrow aspiration were included as the study subjects, and neutrophils, ELA2, and Cit-H3 were detected in the study subjects using ELISA. The blood routine data of 99 patients diagnosed with NB were collected, and these data were from patients different from those of the aforementioned 39 serum samples. And patients were divided into two groups, namely NB patients with positive bone marrow metastasis and those without metastasis, based on the result of bone marrow GD2 immunohistochemical staining. All data were statistically analyzed using IBM SPSS Statistics 26.0 and GraphPad Prism 9.0 software.

**Results:**

A positive correlation was identified between NB bone marrow minimal residual disease (MRD) positivity and haematological indices, including interleukin-2 (IL-2) (*p* = 0.021), IL-6 (*p* = 0.030), NSE (*p* < 0.001), and Cit-H3 (*p* < 0.001). And MRD was positively associated with blood neutrophils in bone marrow (*p* < 0.05).

**Conclusions:**

This study demonstrates a significant correlation between NETs and MRDs, and found a positive correlation between bone marrow MRD^+^ and the indicators of IL-2, IL-6, and NSE, as well as a positive correlation with bone marrow neutrophils. And Cit-H3 was identified as a new biomarker and found a positive correlation between Cit-H3 and bone marrow MRD^+^.

## Background

Neuroblastoma (NB) is the most prevalent extracranial solid tumor in pediatric patients, with the highest mortality rate in children 0–14 years old. Approximately half of NB cases will result in recurrence, metastasis, or progression, with an adverse prognosis [1]. Consequently, this particular tumor lacks effective treatment options [2].

Minimal residual disease (MRD) is the residual tumor cells that persist in patients after local and systemic treatment, and its activation can result in tumor metastasis and recurrence, which is a major challenge for NB patients [3, 4]. Following systemic cancer treatment, residual cancer cells exist as cancer stem cells (CSCs) in primary tumors, circulating tumor cells (CTCs) in peripheral blood, disseminated tumor cells (DTCs) in the bone marrow and lymph nodes, and micrometastases in other metastatic tissues [5]. Early detection of tumor metastasis and recurrence is essential to obtain the best prognosis. The current methods for tumor monitoring in clinical practice are predominantly based on imaging and tumor marker detection. However, invasive tumor biopsy of primary, metastatic, and recurrent tumors are not always feasible. Therefore, the detection and analysis of circulating and disseminated tumor cells by less invasive sampling of peripheral blood and bone marrow has been proven to have clinical relevance in many cancer types, especially in breast, colon, and prostate cancer [6]. Furthermore, circulating tumor DNA in peripheral blood has been shown to be a valuable diagnostic and prognostic marker for various cancers [7].

The unique tumor microenvironment is caused by tumor cells through paracrine and other communication mechanisms. Among the various components, immune cells are the most functional and complex participants, with the capacity to not only kill tumor cells but also promote metastasis [8, 9]. Cytokines, a group of molecules that facilitate interaction between cells, regulate a variety of biological processes, including cell growth, differentiation, maturation, immune response, inflammation, wound healing, and tumor growth and regression. Notably, interleukin-2 (IL-2) [9] and IL-6 [10] play a vital role in the process of antitumor. IL-2 exerts a dual role in tumors, including both antitumor and protumor effects. It has been demonstrated to regulate the development and homeostasis of regulatory T cells, in addition to participating in the differentiation of helper T cells, cytotoxic T cells, and memory T cells [11]. IL-6 has been demonstrated to influence tumor initiation and progression by affecting a number of factors, including but not limited to: tumor cell survival, proliferation, invasion, and the tumor microenvironment [12, 13]. Neutrophils can eliminate invading microorganisms through a variety of mechanisms, including phagocytosis, degranulation, production and release of reactive oxygen species (ROS), and release of neutrophil extracellular traps (NETs) following intense or protracted activation [14]. NETs are a reticular chromatin structure, decorated with histones, particle-derived proteases, and antibacterial peptides. Besides, NETs can directly capture microorganisms through these antibacterial structures, preventing their spread, and local high-concentration antibacterial agents can kill the captured microorganisms [15]. Since the first report in 1996 [16], the release of NETs and subsequent cell death have been the focus of much research [15, 17–20], but the components and factors that determine or promote cell death are still unknown. Neutrophil elastase (ELA2) and citrullinated histone H3 (Cit-H3) have been identified as specific markers of NETs [21]. Elastase 2 is a key protease synthesized by neutrophils themselves, and abnormalities in its function directly affect the life cycle of these cells [21]. Cit-H3 has been found to be associated with the activation and consumption of platelets, and elevated levels of Cit-H3 are frequently accompanied by thrombocytopenia [22]. ELA2 and Cit-H3 can reflect the tumor microenvironment of NB to a certain extent. On the other hand, neuron-specific enolase (NSE) is an enolase involved in the glycolytic pathway, which is present in neural tissues and neuroendocrine tissues. It is also a molecular marker with significant clinical application value, and can also be used as a tumor marker of children’s NB, which can be used to monitor the disease, therapeutic response and predict the recurrence of NB. NETs are a “double-edged sword“ [23]. NETs have been found to control infection [22], yet concurrently, they also contribute to the development and metastasis of cancers such as gastric cancer [24], and this study focuses on whether NETs affect bone marrow metastasis of NB.

The current methods for detecting MRD in NB bone marrow include the bone marrow GD2^+^ immunocytochemical staining technique and the bone marrow GD2^+^ flow detection technique [25]. The former technique will yield red positive results by staining the surface of the NB cell membrane. Conversely, the bone marrow GD2^+^ flow detection technology screens for CD45^−^CD81^+^CD56^+^GD2^+^ cells to identify NB cells. However, it should be noted that there are still some defects in the detection of bone marrow GD2^+^, for example, the sensitivity is only 1/10^5^ and the collected samples are heterogeneous. Moreover, the fact that NB is most prevalent in children further complicates the situation, as it is difficult to collect enough samples for follow-up experiments, and the number of cells collected is often minimal, which may hinder the successful detection of GD2^+^ cells.

It has been shown that children diagnosed with NB frequently exhibit recurrent symptoms of MRD, accompanied by an increase in the number of neutrophils in bone marrow fluid [26, 27]. Based on the above content, we hypothesized that IL-2, IL-6, ELA2, Cit-H3, NSE, and other factors may be associated with bone marrow metastasis of NB. Therefore, this study investigated the correlation between NETs and NB bone marrow MRD by detecting the related indexes of peripheral blood and bone marrow fluid, and aimed to identify specific targets that can determine the occurrence of bone marrow metastasis in NB patients.

## Materials and methods

### Patients and samples

The study collected 99 patients diagnosed with NB by pathological biopsy at Tianjin Medical University Cancer Hospital between 2022 and 2023, including 54 males and 45 females. And the peripheral blood routine data of these 99 patients were collected from Department of Pediatric Oncology, Tianjin Medical University Cancer Institute and Hospital. Bone marrow MRD^+^ is 55 cases and MRD^−^ is 44 cases. Additionally, bone marrow samples were collected from 39 patients diagnosed with NB by postoperative pathological bone marrow puncture from January 2023 to December 2023, including 20 males and 19 females. Bone marrow MRD^+^ is 17 cases and MRD^−^ is 22 cases. All samples were collected at diagnosis, and bone marrow was obtained by clinicians via puncture at the inferior aspect of the tibial tuberosity or the posterior superior iliac spine. The research method conforms to the standards of the Helsinki Declaration. Written informed consents were obtained from each patient, and the study was approved by the local ethics committee (bc2022216).

### MRD detection

According to the consensus criteria of the International Neuroblastoma Risk Group (INRG) Task Force [28], GD2^+^ in bone marrow immunohistochemical staining is defined as MRD^+^.

### Analysis of peripheral blood samples

Routine peripheral blood tests were performed using the Sysmex XN-1000 automatic hematology analyzer with its matching reagents. Coagulation parameters were detected using the Sysmex CS5100 automatic coagulation analyzer and the corresponding supporting reagents.

### Bone marrow neutrophil detection

The content of neutrophils in bone marrow was detected by a full-automatic hematology analyzer. The reference range of the absolute value of neutrophils in children’s bone marrow is (5–12) x10^9^/L, with the relative value of neutrophils set as 40%–75%.

### Detection of Cit-H3 in bone marrow supernatant

The contents of Cit-H3 in bone marrow supernatant were detected by commercial assay kits (Ruixin Biotechnology, Ltd., Quanzhou, China and Boster Biological Technology, Ltd, Wuhan, China respectively) according to the manufacturer’s protocols. In the microplate pre-coated with anti-Cit-H3 antibodies, add Cit-H3 calibrators and test samples, followed by horseradish peroxidase (HRP)-conjugated Cit-H3 antibodies to form a solid-phase antibody-antigen-enzyme-conjugated antibody sandwich complex. Add the substrate, which produces a blue product under the catalysis of HRP. The absorbance (OD value) is measured at a wavelength of 450 nm using a microplate reader, and the OD value is positively correlated with the concentration of Cit-H3 in the test samples. By fitting the calibrator curve, the concentration of Cit-H3 in the samples can be calculated. The detection limit of this method is 0.1 ng/mL.

### Detection of ELA2 in bone marrow supernatant

The contents of ELA2 in bone marrow supernatant were detected by commercial assay kits (Ruixin Biotechnology, Ltd., Quanzhou, China and Boster Biological Technology, Ltd, Wuhan, China respectively) according to the manufacturer’s protocols. After the samples and biotin-conjugated antibodies are sequentially added to the microplate wells for reaction, wash the wells and then add peroxidase-conjugated avidin for reaction. The substrate TMB is converted to a blue product under the catalysis of peroxidase, and further converted to the final yellow product upon the addition of acid. The intensity of the yellow color is positively correlated with the concentration of ELA2 in the samples. The detection limit of this method is 780 pg/mL.

### Statistical analysis

All data, including count, quantitative and ordinal data, were statistically analyzed with IBM SPSS Statistics 26.0. The normal distribution test was conducted on the quantitative data, and the measurement data subject to normal distribution were presented by mean ± standard deviation (X ± SD). The independent sample t-test was used to compare groups. Pearson correlation analysis was conducted to evaluate associations between variables. For quantitative data that did not obey the normal distribution, the median and interquartile interval (M (P25, P75)) were performed and the Mann-Whitney test was used to compare groups. Spearman’s correlation analysis was employed for analyzing correlations involving non-normally distributed or ordinal variables. The chi-square test was used to compare two categories of count data. *p* < 0.05 was considered statistically significant.

## Results

### Clinicopathological characteristics of peripheral blood in NB patients with bone marrow MRD^+^

In this study, the peripheral blood routine data of 99 patients diagnosed with NB by pathological puncture was analyzed. The median concentration of peripheral hemoglobin in patients with MRD^+^ was 89.00 (83.00, 104.75) g/L (Table 1). The platelet volume distribution width was 9.650 (8.500, 12.225) fL (Table 1). Additionally, the median concentration of D-dimer was measured at 1195.1050 (737.9175, 4016.4625) mg/L, and plasma fibrinogen was recorded as 3.2650 (2.5825 g/L, 5.0675) g/L (Table 2). In addition, the median concentration of NSE was 184.2250 (21.5125, 712.2700) ug/mL (Table 3). While patients with MRD^−^ exhibited a median concentration of 103.50 (93.25, 116.50) g/L for hemoglobin in peripheral blood, a platelet volume distribution width median of 10.700 (9.500, 15.775) fL (Table 1), a median concentration of D-dimer of 413.8400 (252.8000, 952.5400) mg/L, a median concentration of plasma fibrinogen of 2.4400 (2.1800, 3.0600) g/L (Table 2), and a median concentration of NSE of 13.0750 (6.6325,20.7325) ug/mL (Table 3). So compared with MRD^−^ patients, MRD^+^ patients exhibited reduced peripheral blood hemoglobin concentration, and platelet volume distribution width levels; whereas, D-dimer, plasma fibrinogen and NSE concentrations were elevated.

**Table 1:** Clinicopathological characteristics of peripheral blood in NB patients

	MRD^+^	MRD^−^	Z	*p*
Erythrocyte (x10^12^/L)	4.705(2.668, 6.602)	5.005(3.495, 8.460)	−1.184	0.236
Hemoglobin (g/L)	89.00(83.00, 104.75)	103.50(93.25, 116.50)	−2.983	0.003^**^
Red blood cell volume distribution width SD (fL)	48.100(43.100, 54.825)	46.95(43.350, 52.100)	−0.647	0.518
Red blood cell volume distribution width CV (%)	15.300(13.025, 17.375)	14.45(13.200, 16.050)	−1.162	0.245
Neutrophil (x10^9^/L)	2.375(1.470, 3.968)	2.565(1.678, 5.015)	−0.853	0.394
Eosinophil (x10^9^/L)	0.0400(0.0100, 0.1100)	0.0600(0.0225, 0.1775)	−1.945	0.052
Basophil (x10^9^/L)	0.0200(0.0100, 0.0375)	0.0002(0.0100, 0.0300)	−0.098	0.922
Lymphocyte (x10^9^/L)	0.9900(0.5075, 1.8600)	1.3200(0.6675, 2.4225)	−1.452	0.146
Monocyte (x10^9^/L)	0.4350(0.1950, 0.5875)	0.4600(0.3500, 0.7175)	−1.629	0.103
Platelet (x10^9^/L)	202.5(126.5, 283.0)	245.0(137.0, 314.0)	−0.956	0.339
Platelet volume distribution width (fL)	9.650(8.500, 12.225)	10.700(9.500, 15.775)	−2.409	0.016^*^

*p<0.05. **p<0.01

**Table 2:** Coagulation characteristics of NB patients

	MRD^+^	MRD^−^	Z	*p*
D-dimer (mg/L)	1195.1050(737.9175, 4016.4625)	413.8400(252.8000, 952.5400)	−3.150	0.002^**^
Thrombin time (s)	16.450(15.425, 16.975)	16.300(15.800, 17.200)	−0.921	0.357
Prothrombin time (s)	12.200(11.425, 12.925)	11.900(11.500, 12.600)	−0.663	0.507
INR	1.0500(0.9825, 1.1175)	1.0300(0.9800, 1.0600)	−0.762	0.446
Activated partial thromboplastin time (s)	27.150(25.025, 32.575)	29.400(26.500, 33.900)	−1.818	0.069
Fibrinogen (g/L)	3.2650(2.5825, 5.0675)	2.4400(2.1800, 3.0600)	−3.028	0.002^**^

*INR* International normalized ratio

**p<0.01

**Table 3:** Tumor markers of peripheral blood in NB patients

	MRD^+^	MRD^−^	Z	*p*
Glu (mmol/L)	1.2900(0.6000, 1.6000)	1.3450(1.0025, 1.8075)	−0.867	0.386
LDH (U/L)	198.550(109.675, 745.125)	151.050(95.600, 188.150)	−0.934	0.351
NSE (ug/mL)	184.2250(21.5125, 712.2700)	13.0750(6.6325, 20.7325)	−3.580	<0.001^***^
Ferr (ug/L)	4187.7300(768.3050, 18852.7075)	3145.9700(781.5825, 4619.4300)	−0.099	0.921

*Glu* Glucose, *LDH* Lactic dehydrogenase, *NSE* Neuron-specific enolase, *Ferr* Ferritin

***p<0.001

### Clinicopathological characteristics of bone marrow in NB patients with bone marrow MRD^+^

In this study, the clinicopathological characteristics of bone marrow in NB patients were analyzed. For NB patients with MRD^+^, the median concentration of IL-2 was 500.00 (434.50, 518.25) pg/mL and the median concentration of IL-6 was 57.73 (7.79, 2495.65) pg/mL (Table 4). While for patients with MRD^−^, the median concentrations of IL-2 and IL-6 in the bone marrow supernatant were 306.00 (218.25,334.50) pg/mL and 10.17 (2.88,13.77) pg/mL, respectively (Table 4). Compared to MRD^−^ patients, the levels of IL-2 and IL-6 in bone marrow supernatant of MRD^+^ patients were increased significantly.

**Table 4:** Cytokines of peripheral blood in NB patients

	MRD^+^	MRD^−^	Z	*p*
IFN-r (pg/mL)	226.50(223.75, 242.00)	247.50(167.00, 256.00)	−0.866	0.386
TNF-a (pg/mL)	328.50(280.50, 445.50)	297.00(173.25, 357.00)	−1.155	0.248
IL-2 (pg/mL)	500.00(434.50, 518.25)	306.00(218.25, 334.50)	−2.309	0.021^*^
IL-4 (pg/mL)	235.50(223.75, 269.75)	201.50(122.50, 235.50)	−1.443	0.149
IL-6 (pg/mL)	57.73(7.79, 2495.65)	10.17(2.88, 13.77)	−2.174	0.030^*^
IL-10 (pg/mL)	272.00(259.75, 309.00)	267.50(181.00, 417.75)	−0.289	0.773
IL-17 A (pg/mL)	205.50(170.25, 241.50)	223.00(119.25, 261.50)	−0.289	0.773

*IFN-γ* interferon-γ, *TNF-α* tumor necrosis factor-α, *IL-2* Interleukin-2

*p<0.05

**Fig. 1:**
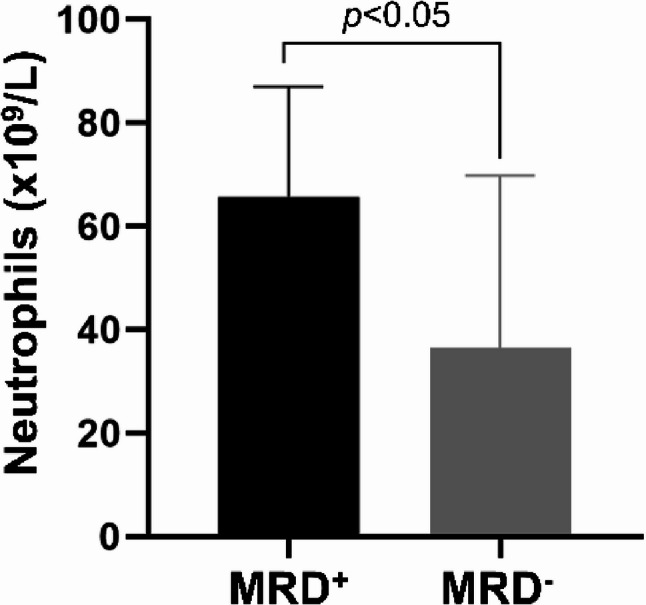
Neutrophils in bone marrow of NB patients. The bone marrow neutrophil count of NB patients was detected by a full-automatic hematology analyzer

In addition, the bone marrow neutrophil count of NB patients with MRD^+^ was 74.3 (44.95, 82.45) × 109/L. In contrast, that of patients with MRD^−^ was 24.64 (11.72, 57.80) × 109/L (Fig. 1). These findings indicate that MRD^+^ patients exhibited a significant augmentation in the neutrophils within bone marrow fluid.

### Concentration of Cit-H3 and ELA2 in bone marrow supernatant of NB patients with bone marrow MRD^+^

Given the evidence that there was a significant increase in neutrophil count in the bone marrow fluid of NB patients with MRD^+^, the contents of Cit-H3 and ELA2 were detected in bone marrow supernatant. The results showed that the concentration of Cit-H3 in bone marrow supernatant of NB patients with MRD^+^ was 78.4000 (62.4450,109.1050) ng/mL, while that of patients with MRD^−^ was 36.6000 (29.7950,43.3625) ng/mL (Table 5). Compared with NB patients with MRD^−^, the level of Cit-H3 in bone marrow supernatant of MRD^+^ patients was significantly higher. In contrast, the median concentration of ELA2 was not significantly different between MRD^+^ and MRD^−^ groups (Table 5). These findings suggest that Cit-H3 is significantly elevated in the bone marrow supernatant of MRD^+^ NB patients.

**Table 5:** Concentration of Cit-H3 and ELA2 in bone marrow supernatant of NB patients

	MRD^+^ (*n* = 17)	MRD^−^ (*n* = 22)	Z	*p*
Cit-H3 (ng/mL)	78.4000(62.4450, 109.1050)	36.6000(29.7950, 43.3625)	−4.956	<0.001^***^
ELA2 (ng/mL)	1046.990(668.865, 1613.390)	1382.475(1007.540, 4754.330)	−1.699	0.089

*Cit-H3* Citrullinated histone H3, *ELA2* Neutrophil elastase

***p<0.001

### Correlation analysis of Cit-H3 and GD2% in bone marrow supernatant of NB patients with bone marrow MRD^**+**^

To identify a specific marker for evaluating the presence of bone marrow metastasis in NB patients, those with bone marrow MRD^+^ were divided into four distinct groups according to quartiles of GD2% in bone marrow MRD^+^ cases: I (0–25%), II (25–50%), III (50–75%) and IV (75–100%). By non-parametric analysis, no statistical significance was found between Cit-H3 concentration in bone marrow plasma and GD2% in high-risk NB patients with MRD^+^ (Table 6). These findings suggest that Cit-H3 was independent of the GD2^+^ particles in MRD^+^ NB patients.

**Table 6:** Correlation analysis of Cit-H3 and GD2% in bone marrow supernatant of NB patients

Sample (*n* = 15)	Group	GD2^+^ particle	GD2^+^/GD45^+^	Cit-H3 (ng/mL)	*p*
1	Ⅳ	7716	10.80%	93.13	0.83
2	Ⅲ	1944	1.19%	70.39	
3	Ⅱ	438	0.32%	129.07	
4	Ⅰ	29	0.14%	53.81	
5	Ⅱ	166	0.17%	89.45	
6	Ⅱ	139	0.12%	62.28	
7	Ⅳ	29,406	5.45%	62.61	
8	Ⅳ	22,024	8.35%	194.96	
9	Ⅰ	76	0.01%	86.62	
10	Ⅰ	9	0.00%	78.40	
11	Ⅲ	3134	0.83%	204.54	
12	Ⅰ	46	0.12%	76.12	
13	Ⅲ	456	1.09%	45.37	
14	Ⅲ	775	0.72%	109.23	
15	Ⅱ	206	1.43%	71.17	

*Cit-H3* Citrullinated histone H3

### Analysis of serological indicators in the diagnosis of bone marrow MRD^+^ in NB patients

**Fig. 2:**
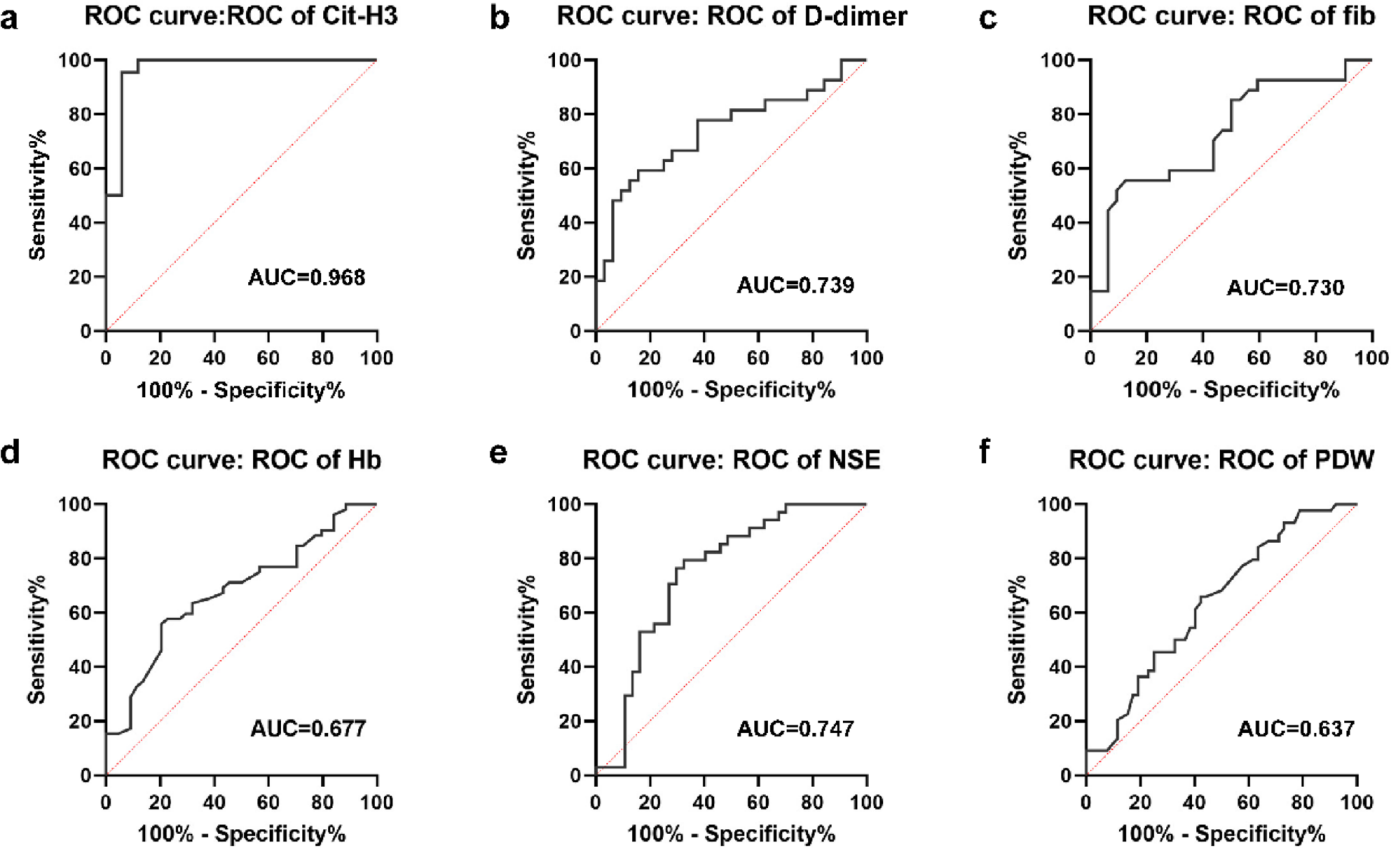
ROC analysis of MRD^+^ patients diagnosed by different serological indicators. ROC curve is used to evaluate the diagnostic value of bone marrow Cit-H3 (**A**), peripheral blood D-dimer (**B**), fibrinogen (**C**), hemoglobin (**D**), NSE (**E**) and platelet volume distribution width (**F**) as bone marrow MRD^+^ in NB patients

To evaluate the diagnostic value of peripheral blood hemoglobin, the receiver operating characteristic (ROC) curve was constructed, platelet volume distribution width, D-dimer, plasma fibrinogen, NSE and bone marrow Cit-H3 as bone marrow MRD^+^ in NB patients. The results showed that the area under the curve (AUC) of bone marrow Cit-H3 was 0.968, with a cut-off value of 45.32 ng/mL (Fig. 2).

Additionally, the binary Logistic regression analysis of serological indicators showed that Cit-H3 in bone marrow functioned as an independent influencing factor of bone marrow MRD^+^ in NB patients (Table 7), while the width of platelet volume distribution and the content of D-dimer were not independent influencing factors (Table 8). These findings suggest that Cit-H3 and MRD^+^ are positively correlated, and Cit-H3 can be a novel biomarker of MRD in NB patients.

**Table 7:** Logistics regression analysis of bone marrow indicators

	β	SE	Waid	*p*	OR	95%CI
Sex	−0.706	1.097	0.414	0.52	0.494	0.057 ~ 4.238
Age	0.74	1.167	0.402	0.526	2.097	0.213 ~ 20.658
Cit-H3	0.131	0.059	4.968	0.026^*^	1.14	1.016 ~ 1.28

*Cit-H3* Citrullinated histone H3

*p<0.05

**Table 8:** Logistics regression analysis of peripheral blood indicators

	β	SE	Waid	*p*	OR	95%CI
Sex	0.695	0.558	1.549	0.213	2.003	0.671 ~ 5.98
Age	−0.589	0.611	0.93	0.335	0.555	0.167 ~ 1.838
Platelet volume distribution width	0.039	0.1	0.149	0.7	1.039	0.854 ~ 1.264
D-dimer	0	0	2.538	0.111	1	1 ~ 1

## Discussion

NB is the most prevalent extracranial solid tumor among pediatric patients, accounting for 8%−10% of childhood malignant neoplasms with a mortality rate of 15% [1]. In this study, we analyzed the clinicopathological characteristics of peripheral blood of NB patients with bone marrow MRD^+^. Compared to MRD^−^ NB patients, those patients with MRD^+^ showed significant alterations in peripheral blood, hemoglobin, platelet volume distribution width, D-dimer and plasma fibrinogen, suggesting that these indicators could serve as valuable tools for evaluating bone marrow MRD metastasis in NB patients. Monitoring these parameters is of great significance for the timely identification of bone marrow metastasis in NB patients. Subsequent analysis of cytokines and additional indicators within the immune microenvironment revealed that MRD^+^ NB patients showed significantly elevated levels of IL-2 and IL-6 in bone marrow supernatant and NSE in peripheral blood when compared to MRD^−^ patients. Moreover, a positive correlation was identified between bone marrow MRD^+^ and neutrophil level. Additionally, the detection of Cit-H3 and ELA2, the specific targets of NETs, were detected, and a positive correlation between bone marrow MRD^+^ and Cit-H3 was observed, suggesting a potential role for NETs in the bone marrow metastasis of NB. Logistic regression analysis further indicated that Cit-H3 was an independent influencing factor of bone marrow MRD^+^ in NB patients.

NSE, an enolase involved in the glycolytic pathway, exists in both nerve tissue and neuroendocrine tissue. Researches showed that an evaluated NSE was associated with neuroendocrine tumors. This increase has been observed in patients diagnosed with small cell lung cancer (SCLC), along with the elevated level of NSE in serum. In NB patients, the positive rate of NSE can be as high as 96%−100% [29, 30], with the serum NSE level correlating to the clinical stage and the subsequent prognosis of the disease. Recent studies have indicated that the combination of serum carbohydrate antigen 125 (CA-125), NSE and 24-hour urinary vanillylmandelic acid (VMA) may be more effective in predicting the recurrence of NB [31]. Zeltzer et al. found that elevated serum NSE level (above 100 ng/mL) was associated with poor outcomes for patients with extensive metastatic NB (stage IV) [32, 33]. Isgro et al. found that the NSE levels of 137 NB patients were all above 100 ng/mL before treatment, all below 100 ng/mL in remission, and all above 100 ng/mL in serum during relapse [34]. Similarly, our study showed that the concentration of NSE in peripheral blood of NB patients with MRD^+^ is significantly increased. Thus, NSE can be used as an important indicator to detect bone marrow MRD metastasis in NB patients.

IL-2, a pleiotropic cytokine, plays an important role in the immune system. It exerts a “contradictory” biological activity, exhibiting both immune stimulation and immunosuppression [8], and the precise influence of IL-2 on non-hematopoietic and innate immune cells is still unclear. Research has demonstrated that IL-2 can regulate the development of T cells and induce the proliferation of CD8^+^ and CD4^+^ T cells. In addition, IL-2 exerts a number of other biological activities, such as increasing cell lysis activity, promoting antibody production and B cell proliferation. Furthermore, there are also studies on the fusion of two IL-2 molecules into monoclonal antibodies that recognize GD2 [35, 36]. The preclinical and initial clinical data showed that the curative effect is better in MRD^+^ patients, indicating that IL-2 is a promising therapeutic agent for NB patients.

IL-6, a pivotal regulator of inflammation, autoimmunity and cancer, predominantly exerts its function via the IL-6/STAT3 pathway [13, 37]. The crosstalk between tumor cells and their microenvironment is critical in tumor formation and progression. Siltuximab, an anti-IL-6 antibody, has demonstrated significant therapeutic benefits in the management of diverse human cancers, whether administered as a monotherapy or in combination with other chemotherapy agents [16, 38]. In this study, the high expression of IL-6 in patients with MRD^+^ suggests a potential role for IL-6 in the process of bone marrow metastasis of NB, and the promotion of immune escape by tumor cells, which is expected to become a novel biomarker for monitoring bone marrow metastasis of NB in the future.

NETs are reticular structures secreted by activated neutrophils, which composed of DNA, histone and antibacterial protein, and are responsible for capturing and killing extracellular pathogens. Neutrophils, vital for sterilization, undergo a process of programmed cell death when specifically stimulated. This results in the release of DNA within cells, which then forms a network structure alongside other substances, effectively trapping bacteria for further elimination. The increase of neutrophils in NB patients with MRD^+^ in bone marrow suggests the elevated NET formation. Therefore, we further detected the special targets Cit-H3 and ELA2 of NETs. The results showed that Cit-H3 in bone marrow of NB patients with MRD^+^ was significantly higher than that of patients with MRD^−^, but ELA2 was not significantly changed. Recent studies have indicated that immunotherapy targeting GD2, a marker expressed on tumor cells by the use of T cells expressing chimeric antigen receptors, holds potential as a treatment option for patients with high-risk neuroblastoma [36]. We therefore analyzed the correlation between GD2 and Cit-H3. The lack of correlation between Cit-H3 levels and GD2^+^ particles suggests that Cit-H3 may not reflect tumor burden directly; however, it could still serve as an independent marker of immune activity. Nevertheless, the detection of NETs has the potential to not only provide a new treatment target but also provide a new screening method, thereby facilitating early diagnosis and treatment.

The serological indicators of peripheral blood and bone marrow fluid were analyzed by constructing the ROC curve and binary Logistic regression analysis. It was found that Cit-H3 exhibited a high AUC value, with a cut-off value of 45.32 ng/mL, suggesting that bone marrow Cit-H3 can used as an independent influencing factor for diagnosing bone marrow MRD^+^ in NB patients.

Nevertheless, due to the rarity of this disease, the number of cases in this study is limited. On the other hand, we did not obtain multivariate results and relied exclusively on univariate comparisons without controlling for confounding factors such as age, stage, or treatment status—this may be a potential reason for the lack of statistically significant changes in ELA2 levels among MRD^+^ NB patients. Meanwhile, it is also necessary to expand the sample size for further verification.

## Conclusions

In summary, this study highlights the correlation between NETs and bone marrow MRD^+^, aiming to identify a specific target to determine the occurrence of bone marrow metastasis in NB patients. In addition, a positive correlation between Cit-H3 and bone marrow MRD^+^ is observed. Compared with the traditional methods for detecting bone marrow GD2, this study selected bone marrow supernatant as the detection sample, thereby circumventing the limitations associated with sample heterogeneity and small sample size. The combined detection of bone marrow Cit-H3 and GD2 holds promise as a future diagnostic tool for identifying bone marrow metastasis in NB patients.

## Data Availability

The datasets used and/or analyzed during the current study are available from the corresponding author on reasonable request.

## Abbreviations

Cit-H3: Citrullinated histone H3
CSCs: Cancer stem cells
CTCs: Circulating tumor cells
DTCs: disseminated tumor cells
ELA2: Neutrophil elastase
MRD: Minimal residual disease
NB: Neuroblastoma
NETs: Neutrophils release neutrophil extracellular traps
NSE: Neuron-specific enolase
ROS: Reactive oxygen species
